# Inhibition of epidermoid carcinoma A431 cell growth and angiogenesis in nude mice by early and late treatment with a novel dextran derivative

**DOI:** 10.1038/sj.bjc.6600985

**Published:** 2003-06-10

**Authors:** M Di Benedetto, A Starzec, R Vassy, G Y Perret, M Crépin, M Kraemer

**Affiliations:** 1 Laboratoire d'Oncologie Cellulaire et Moléculaire, UPRES 2360, Université Paris 13, 74 rue Marcel Cachin, 93017 Bobigny cedex, France; 2 Laboratoire de Pharmacologie, UPRES 2360, Université Paris 13, 74 rue Marcel Cachin, 93017 Bobigny cedex, France; 3 Laboratoire d'Hémostase, Endothélium et Angiogénèse, Unité INSERM 553, Hôpital Saint-Louis, 75010 Paris, France

**Keywords:** tumour angiogenesis, phenylacetate carboxymethyl benzylamide dextran (NaPaC), aponecrosis, vascular endothelial growth factor (VEGF)

## Abstract

We investigated the effect of a new dextran derivative, phenylacetate carboxymethyl benzylamide dextran (NaPaC), on epidermoid carcinoma A431 cells secreting a large quantity of angiogenic factor, vascular endothelial growth factor (VEGF). *In vitro*, NaPaC inhibited the proliferation of A431 cells (IC_50_=5 *μ*M). Also, NaPaC decreased the binding of radiolabelled VEGF_165_ to endothelial cells (IC_50_=0.2 *μ*M). *In vivo*, we explored the effects of NaPaC (15 mg kg^−1^) on A431 xenograft growth starting the drug administration at the time of tumour cell inoculation (early treatment) and 1 week later, when tumours were well established (late treatment). Early treatment was more efficient on tumour inhibition (70% *vs* control) than late treatment (50% *vs* control). Early and late NaPaC-treatment increased the aponecrosis in tumour by 70 and 30%, respectively. Whatever treatment, NaPaC inhibited the intratumour endothelial cell density in the same manner. In contrast, vessel area was decreased only when NaPaC was injected early (35%). These results show that NaPaC has a potent inhibitory effect, dependent on treatment outset, on epidermoid carcinoma growth associated with an intratumour microvascular network diminution and an aponecrosis increase. As this drug is nontoxic at efficient dose, it offers interesting perspectives for the therapy of malignant lesions.

Angiogenesis, the formation of new blood vessels from established vessels, occurs under a variety of normal and pathological conditions. Also, it is a requisite for tumour growth and metastasis dissemination (Blood and Zetter, 1990; Ramanujan *et al*, 2000). The delivery of blood-borne nutrients to the tumour cells is essential for their survival and spread. Thus induction of angiogenesis was observed to precede the development of invasive tumours (Weidner *et al*, 1991).

We recently demonstrated *in vitro* that phenylacetate carboxymethyl benzylamide dextran (NaPaC) inhibited the secretion of growth factors from breast cancer cells and prevented the action of growth factors by interacting with them (Di Benedetto *et al*, 2002). In particular, we showed that NaPaC formed complexes with vascular endothelial growth factor (VEGF_165_), which is a specific mitogenic factor for endothelial cells. Vascular endothelial growth factor is the best-characterised VEGF-A form the expression of which has been correlated, temporally and/or spatially, with the onset of angiogenesis in a variety of tumours including lung (Senger *et al*, 1986), breast (Krantz *et al*, 1999), ovarian (Shen *et al*, 2000) and colon cancer (Cascinu *et al*, 2000).

In this report, we investigated the effect of NaPaC on the *in vitro* and *in vivo* growth of epidermoid carcinoma A431 cells that secrete a large amount of VEGF (Myoken *et al*, 1991). First, we explored *in vitro* if NaPaC could inhibit the A431 cell proliferation and prevent the binding of VEGF_165_ on tumour and endothelial cells. Then *in vivo*, we assessed the effects of NaPaC on the A431 tumour growth, cell death and microvascular system development in xenografts implanted in nude mice. Since angiogenesis occurred as specific spatiotemporal events (Mori *et al*, 1999) and since distinct antiangiogenic drugs have been shown to be effective at different stages of tumorigenesis (Bergers *et al*, 1999), we have studied and compared the tumours from animals treated with NaPaC starting at early or late stage of xenograft development.

## MATERIALS AND METHODS

### Dextran derivative preparation

**Figure 1:**
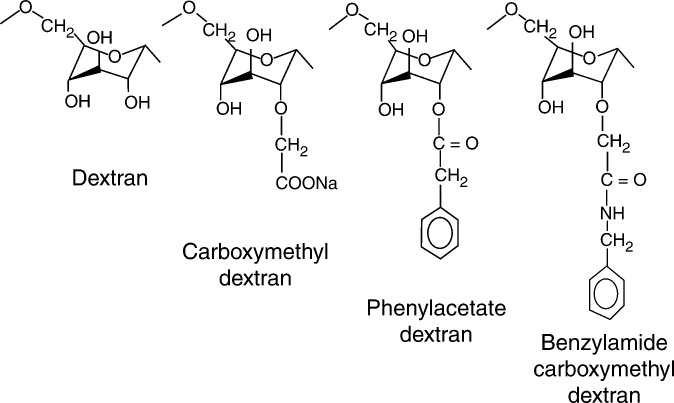
Structure of NaPaC.

New dextran derivative, phenylacetate carboxymethyl benzylamide dextran (Figure 1

), named NaPaC, was synthesised by Biodex Laboratory (Supplier) (Levallois-Perret, France) performing a statistical esterification of carboxymethyl benzylamide dextran with phenylacetic acid (Avramoglou *et al*, 2001). After purification by ultrafiltration (purity >98%) and lyophilisation, the chemical composition or degree of substitution (ds) of NaPaC was determined by acidimetric titration and elementary analysis of nitrogen. The composition of NaPaC was: 0 ds for dextran, 0.67 ds for carboxymethyl and 0.39 ds for benzylamide and a phenylacetate ds of 0.35. The calculated average molecular weight of NaPaC mer was 264.1 gU^−1^. Absolute molecular weight was calculated multiplying the average molecular weight by the number of units (247). This calculation leads to MW: 77 607 g mol^−1^.

### Cell culture

Human epidermoid carcinoma A431 cells and human umbilical vein endothelial cells (HUV-EC) were purchased from American Tissue Culture Collection (Rockville, MD, USA). They were routinely grown in DMEM (Life Technologies, Inc., Gaithersburg, MD, USA), supplemented with 10% FCS, 2 mM L-glutamine, 1 mM sodium pyruvate, 50 U ml^−1^ penicillin and 50 mg ml^−1^ streptomycin (all obtained from Life Technologies, Inc.), at 37°C in a 5% CO_2_-humidified atmosphere.

### Cell growth assays

A431 cell growth was assessed using the MTT-microculture tetrazolium assay (Mosmann, 1983). Briefly, the cells (4 × 10^3^) were incubated in 2% FCS–DMEM for 24 h and then treated with NaPaC at different concentrations for 72 h. Then, the cells were washed with phosphate buffer saline (PBS) and incubated with 0.1 ml of MTT (2 mg ml^−1^) for 4 h.

### Binding competition assay

HUV-EC and A431 cells were grown until 80% confluence in 24-well tissue culture plates (Falcon, Strasbourg, France). After an overnight incubation in serum-free medium and two washings with ice-cold binding buffer (PBS, 0.2% gelatine), cells were incubated at 4°C for 2 h in 0.3 ml of binding buffer containing 7 pM ^125^I-VEGF_165_ (Amersham Pharmacia Biotech, Orsay, France) in the presence or absence of NaPaC at increasing concentrations (0–24 *μ*M). Incubation was arrested by gently removing the medium and washing the cell monolayer three times with ice-cold binding buffer. The radioactivity bound to cells was measured in gamma counter (LKB 1261 Multigamma) after cell lysis in 0.3 ml of 0.5 N NaOH for 30 min. Nonspecific binding was determined in the presence of an excess (5 nM) of unlabelled VEGF_165_ (R&D Systems, Abingdon, UK). For the Scatchard plot analysis (Scatchard, 1986), binding was accomplished with increasing concentrations of unlabelled VEGF_165_ (0–5000 pM) and 7 pM ^125^I-VEGF_165_ in the presence or absence of NaPaC at IC_50_. Each curve was analysed according to the Scatchard procedure or by fitting a logistic curve (Graph Pad Software). All analyses were performed twice and carried out in triplicate.

### Xenografts in nude mice

All *in vivo* experiments were carried out with ethical committee approval and met the standards required by the UKCCCR guidelines (Workman *et al*, 1998). A431 cells (1 × 10^5^) were inoculated subcutaneously (s.c.) near the right mammary fad pad of 4-week-old athymic nude mice (nu/nu, *n*=40) (Harlan Laboratory, Gannat, France). Animals were kept in a temperature-controlled room on a 12 : 12 light–dark schedule with food and water *ad libitum*. Phenylacetate carboxymethyl benzylamide dextran was administrated following two protocols starting at early or late stage of xenograft development. In the first treatment, the administration of NaPaC (15 mg kg^−1^, *n*=10) begun at the time of A431 cell inoculation: the tumour cells were injected s.c. in 0.1 ml of NaPaC solution. In the control group (*n*=10), the A431 cells were injected in 0.1 ml of 0.9% NaCl. The drug or 0.9% NaCl solution was administrated twice a week for 5 weeks. In the other protocol (late), the NaPaC treatment started 1 week after cell inoculation when palpable tumours have been developed. It is noteworthy that these tumours were observed in 20 out of 20 animals. Then, mice were arbitrarily placed in control (*n*=10) and NaPaC-treated group (*n*=10). Phenylacetate carboxymethyl benzylamide dextran (15 mg kg^−1^) was injected in 0.1 ml of 0.9% NaCl s.c. near the tumour, twice a week for 5 weeks. Control received 0.1 ml of 0.9% NaCl. Tumour volumes were calculated as previously described (Di Benedetto *et al*, 2001). In our experiment, we have used NaPaC at doses previously reported to be efficient on breast cancer cells xenografted in nude mice (Di Benedetto *et al*, 2002).

### Endothelial cell staining in tumour sections

Tumour specimens were fixed with a solution of paraformaldehyde (4%) and included into paraffin using standard procedure. Routinely, 5 *μ*m sections were stained in haematoxylin and eosin. For immunohistochemical studies, the sections were deparaffinised and rehydrated. Endogenous peroxidase was inactivated with 3% H_2_O_2_. After washing in TBS (0.05 M Tris, 1.5 M NaCl, pH 7.6), the tumour sections were preincubated with 10% normal goat serum for 1 h at room temperature. Endothelial cells were specifically labelled with GSL-1 isolectin B4 (Vector Laboratories, Burlingame, CA, USA). The GSL-1 lectin binds specifically to galactosyl residues and thus labels the mouse endothelial cells (Alroy *et al*, 1987). The sections were labelled for 1 h with the 1 : 50 diluted GSL-1 isolectin at room temperature, then incubated with goat antibody against GSL-1 isolectin B_4_ (1 : 400 dilution, Vector Laboratories) for 30 min, washed with TBS and incubated with biotinylated rabbit anti-goat immunoglobulins (1 : 400 dilution; Dako, Glostrup, Denmark) for 20 min in a moist chamber at room temperature. After three washes with TBS, samples were incubated with streptavidin–biotin peroxidase (LSAB kit; Dako) for 10 min using 3-amino-9-ethylcarbazole (AEC) chromogen, giving a red staining. Finally, slides were washed in water and counterstained with haematoxylin.

### Microvessel analysis in tumour sections

Intratumour number of endothelial cells per tumour section area (endothelial cell density) was determined using a point-counting grid over the GSL-1-labelled cells (96 points in the grid corresponding to an area of 1.02 mm^2^ on the picture) (Weibel, 1979). For each tumour, 10 randomly selected nonserial sections were studied. For each section, 10 fields containing exclusively viable tumour cells, as indicated by the haematoxylin staining, were selected randomly for analysis. Using a Reichter-Jung (Polivar, Austria) microscope, each tumour was scanned at × 100 magnification to select the regions with the most intense vascularisation following the criteria previously defined (Weidner *et al*, 1991). For each region, at least two pictures were taken at × 250 magnification. The highest number of endothelial cells identified within any × 250 field (1.02 mm^2^) was taken into account. The coefficient of variation (SD) was used to assess the variability of counts divided by field number of the same tumour. Mean intratumour endothelial cell numbers per area in the various tumours were compared using Student's *t*-test. To estimate the area of vessels in tumour section, the lumens bordered with at least one GSL-1-stained endothelial cell were counted using the point-counting grid. The intratumour vessel area was expressed as the ratio of determined counts to total points of grid (96) according to Weibel method (Weibel, 1979). Thus, vessel area represents the fraction of the total tissue area occupied by the wall or lumen and reflects the overall number and size of vessels. For all statistical analyses, the level of significance was set at 0.05.

### Cell death detection and quantification in tumour sections

Tumour sections (5 *μ*m) were deparaffinised and rehydrated, then analysed for cell death DNA fragmentation using TumorTACS kit (R&D Systems, Abington, UK). Intratumour aponecrotic cells were counted using a point-counting grid over the apoptotic cells as described above for endothelial cells. For each tumour section, 10 different fields were selected for analysis.

### Statistical analysis

Multiple statistical comparisons were performed using ANOVA in a multivariable linear model. Some statistical analyses were performed using the Mann–Whitney *t*-test. *P*<0.05 was considered statistically significant.

## RESULTS

### NaPaC inhibits the *in vitro* proliferation of epidermoid carcinoma A431 cells

**Figure 2:**
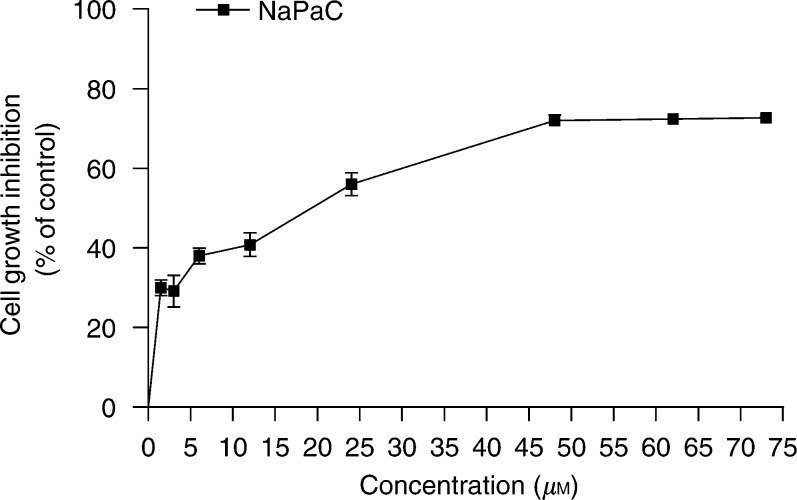
Phenylacetate carboxymethyl benzylamide dextran inhibits the A431 cell proliferation. Cells were incubated for 72 h in the absence or presence of NaPaC at various concentrations. Cell growth was assessed using MTT-assay as described in Materials and Methods. Each point represents the mean ±s.d. of three independent experiments.

We have recently shown that NaPaC has an antiproliferative effect on various breast cancer cells (Di Benedetto *et al*, 2002). Here, we demonstrated that NaPaC is able to inhibit the *in vitro* growth of epidermoid carcinoma A431 cells in a dose-dependent manner (Figure 2

). After a 72 h incubation, the maximal inhibitory effect (70%) was achieved in the presence of 48 *μ*M NaPaC (*P*=0.03). The NaPaC concentration inducing 50% of maximal inhibition (IC_50_) was 5 *μ*M.

### Phenylacetate carboxymethyl benzylamide dextran inhibits VEGF_165_ binding to A431 cells

**Figure 3:**
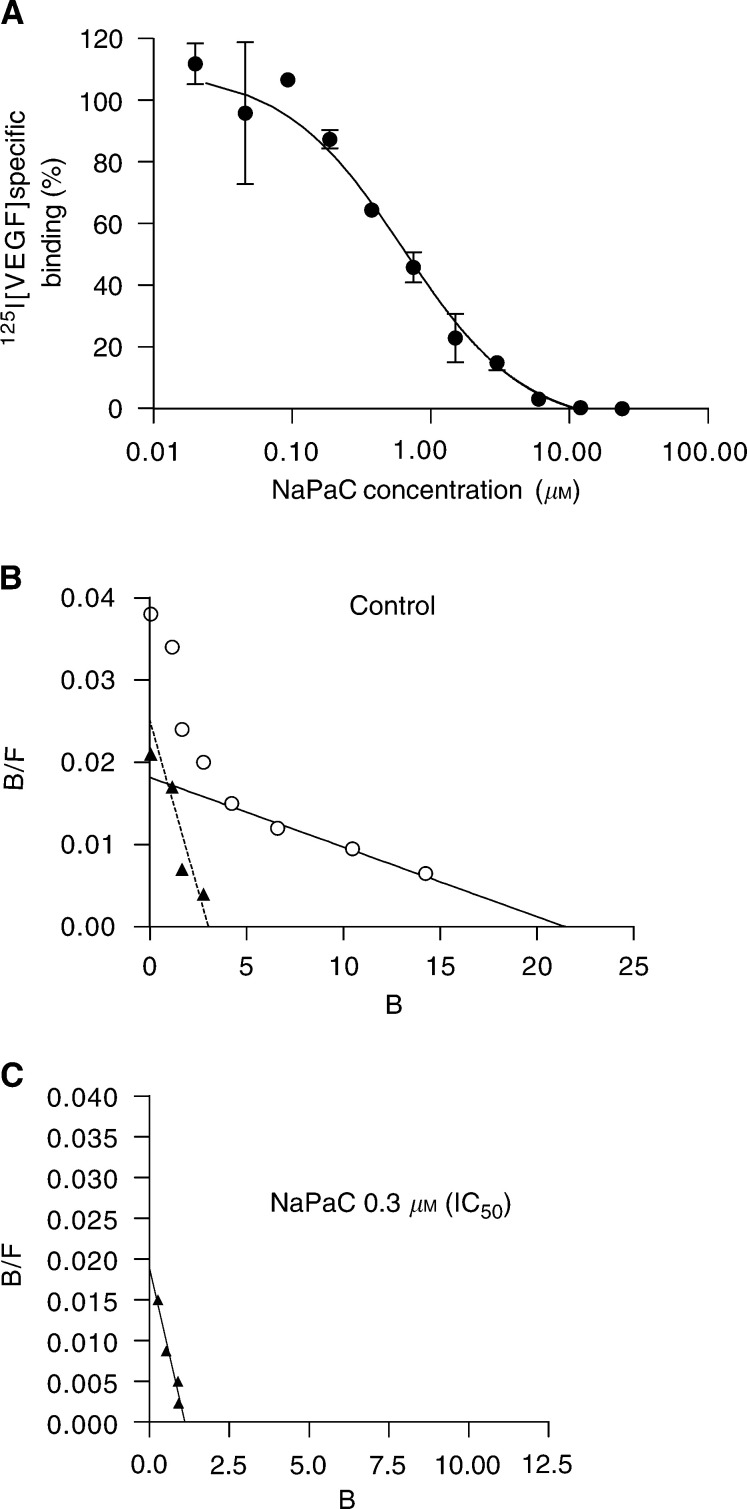
NaPaC inhibits the VEGF_165_ binding to A431 cells. (**A**) Cells were incubated with a fixed concentration of [^125^I]VEGF_165_ (7 pM) in the absence or presence of NaPaC at various concentrations (0.0375–24 *μ*M). (**B**, **C**) Scatchard analysis was performed using 7 pM [^125^I]VEGF_165_ and unlabelled VEGF_165_ at various concentrations in the absence (**B**) or presence (**C**) of 0.3 *μ*M NaPaC (IC_50_).

As we recently showed that NaPaC forms a complex with VEGF_165_ (Di Benedetto *et al*, 2002) and as A431 cells secrete high amounts of VEGF_165_ (Myoken *et al*, 1991) we tested, here, the effect of NaPaC on the binding of VEGF to A431 cells (Figure 3

). Phenylacetate carboxymethyl benzylamide dextran inhibited the binding of VEGF_165_ to A431 cells in a concentration-dependant manner with an IC_50_ of 0.3 *μ*M (Figure 3A). The binding of VEGF_165_ was completely abolished by 6 *μ*M NaPaC. For Scatchard analysis, the cells were incubated with radiolabelled VEGF_165_ (7 pM) and unlabelled VEGF_165_ at increasing concentrations in the presence (Figure 3C) or in the absence (Figure 3B) of 0.3 *μ*M NaPaC (IC_50_). In control conditions (in the absence of NaPaC), two classes of binding sites were observed. The higher affinity class is characterised by a *K*_d_ of 100 pM and the lower affinity population by a *K*_d_ of 1200 pM. The addition of 0.3 *μ*M (IC_50_) NaPaC did not significantly affect the affinity of the first class sites, but induced the disappearance of the low-affinity population (Figure 3C). This can be explained by the fact that NaPaC at IC_50_ formed a complex only with a fraction of VEGF_165_, thus decreasing the concentration of the remaining available growth factor below the level required for binding to low-affinity sites. At higher concentration (6 *μ*M), NaPaC was able to block VEGF_165_ binding to high-affinity sites since no specific binding was observed (Figure 3A). These experiments clearly showed that NaPaC prevented the VEGF_165_ binding to A431 cells involving, at least in part, interactions with the growth factor.

### Phenylacetate carboxymethyl benzylamide dextran inhibits the VEGF_165_ binding to human umbilical vein endothelial cells

**Figure 4:**
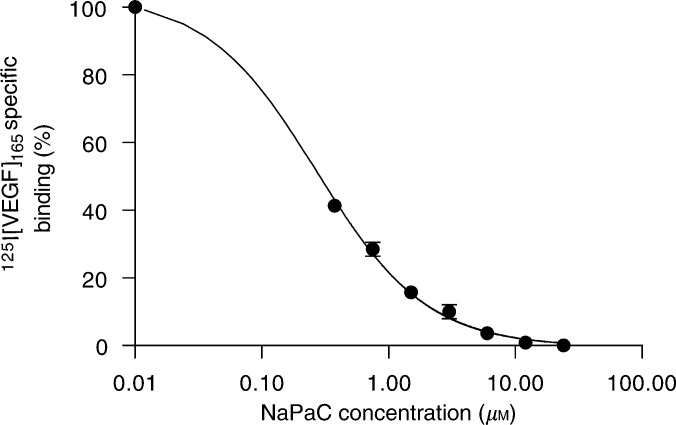
NaPaC inhibits the VEGF_165_ binding to HUV-EC endothelial cells. Cells were incubated with a fixed concentration of [^125^I]VEGF_165_ (7 pM) in the absence or presence of NaPaC at various concentrations (0.01–24 *μ*M)

Phenylacetate carboxymethyl benzylamide dextran inhibited the binding of VEGF_165_ to human umbilical vein endothelial cells (HUV-EC) in a concentration-dependant manner with an IC_50_ of 0.2 *μ*M (Figure 4

). The binding of VEGF_165_ was completely abolished by 6 *μ*M NaPaC. Scatchard analysis revealed in control conditions (in the absence of NaPaC), two classes of binding sites as observed by others (Soker *et al*, 1996; Li *et al*, 2001). The higher affinity class is characterised by a *K*_d_ of 355 pM and the lower affinity population by a *K*_d_ of 1000 pM. The addition of 0.2 *μ*M of NaPaC (IC_50_) did not significantly affect the affinity of the first class sites, but induced the disappearance of the low-affinity population (data not shown). The disappearance of high-affinity sites was achieved in the presence of drug at a higher concentration (6 *μ*M). Like for A431 cells (above), these experiments clearly showed that NaPaC inhibited the VEGF_165_ binding to HUV-EC cells probably by forming a complex with the growth factor.

### Phenylacetate carboxymethyl benzylamide dextran inhibits the A431 xenograft growth more efficiently when administrated early

**Figure 5:**
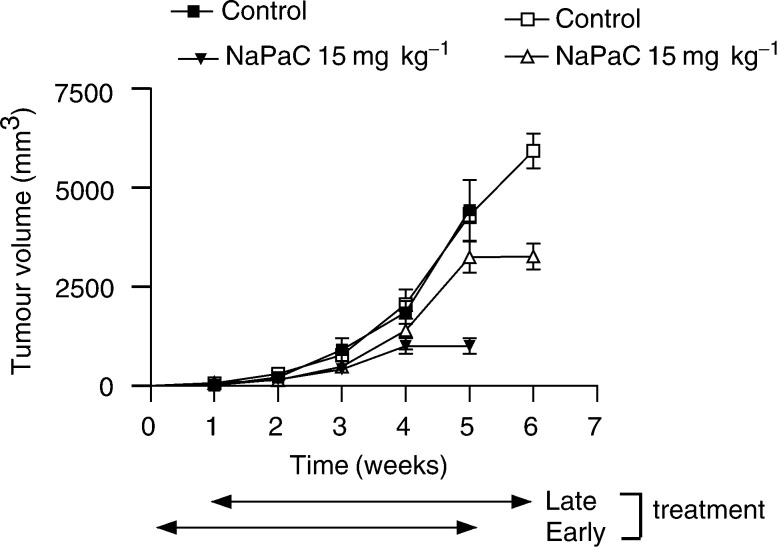
A431 tumour growth inhibition induced by early and late administrations of NaPaC in nude mice. Early treatment (black symbols) was performed by a simultaneous s.c. inoculation of A431 cells (1 × 10^5^) at day 0 and NaPaC (15 mg kg^−1^). Late s.c. treatment (white symbols) with NaPaC (15 mg kg^−1^) began 1 week after tumour uptake, when tumours were well established (=100 mm^3^). NaPaC was injected twice a week for 5 weeks for both early and late treatment. Control groups received 0.1 ml of 0.9% NaCl for the same period. Each point represents the mean of tumour volume (mm^3^) ± s.d. (*n*=10).

We evaluated the A431 xenograft growth when NaPaC administration begun simultaneously with tumour cell inoculation (early treatment, Figure 5

, black symbols) and when NaPaC injection, at the same dose and for the same period of 5 weeks, started 1 week after A431 cell inoculation, when palpable tumours appeared (late treatment, Figure 5, white symbols). Whatever treatment, early or late, a significant inhibition of xenograft growth was observed at the 5th week of NaPaC administration. However, early NaPaC treatment reduced the tumour growth by 70% as compared to control (*P*=0.0067), whereas late administration of the drug inhibited the A431 tumour growth by 50% (*P*=0.0011). Early administration of NaPaC was not able to affect the A431 tumour uptake. The chronic administration of NaPaC (15 mg kg^−1^) to A431 xenograft-bearing mice, twice a week for 5 weeks, did not cause signs of toxicity. The body weight of mice was not affected. No diarrhoea, infection, weakness or lethargy was stated. All of the 40 studied mice were alive at the end of treatments.

### Phenylacetate carboxymethyl benzylamide dextran induces cell death in tumour more effectively when administrated early

**Figure 6:**
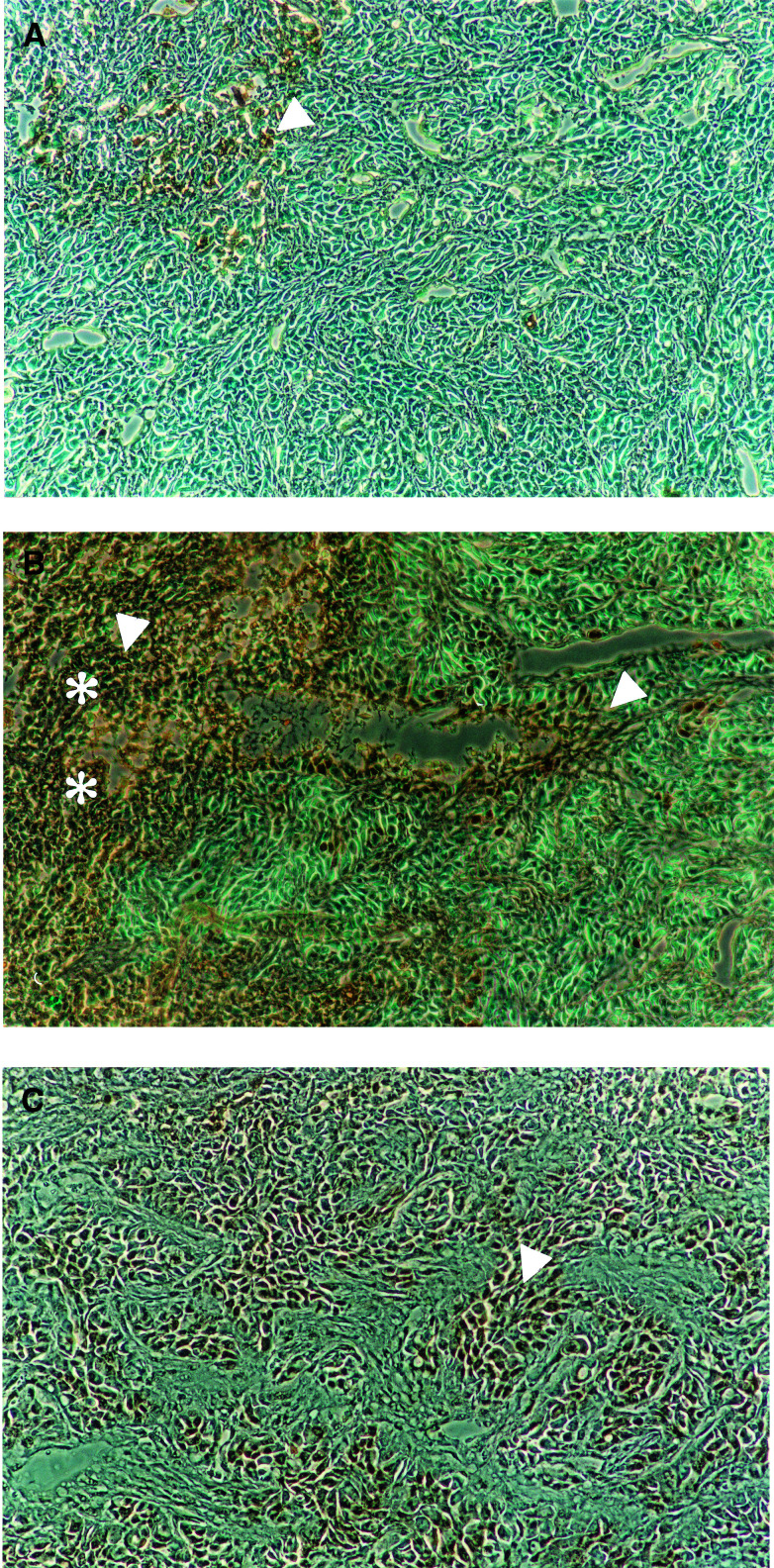
Phenylacetate carboxymethyl benzylamide dextran induces the cell death in early and late treated A431 tumours. Cell death of untreated (**A**) and early (**B**) or late (**C**) treated tumours was assessed by terminal deoxynucleotidyl transferase-mediated nick-end labelling using Tumour TACS kit. Necrotic area was marked with asterisks. Representative aponecrotic cells were marked with arrows.

In both, early (Figure 6B

) and late (Figure 6C), NaPaC-treated tumours, we observed a more intense brown staining of the nuclei of apoptotic cells as well as a more diffused brown staining of the cytoplasm and the nuclei of necrotic cells as compared to control (Figure 6A). Since the difference between the staining of necrotic and apoptotic cells was difficult to distinguish, we counted all brown-stained cells. This statement is in agreement with our recent observations that, in breast cancer xenografts, NaPaC induced rather aponecrosis (Di Benedetto *et al*, 2002) described by Formigli *et al* (2000) than classical apoptosis. In the early treated tumours, large regions of necrosis were observed (Figure 6B) and the number of aponecrotic cells per area was increased by 70% as compared to control (*P*<0.0001). In the case of late treatment with NaPaC, the density of aponecrotic cells was increased by 30% compared to control (*P*<0.0001, Figure 6C *vs* A) and the necrotic regions were diminished as compared to early treated tumours (representative photos shown in Figure 6).

### Effect of early- and late-administrated NaPaC on the microvascular system of A431 tumour

**Figure 7:**
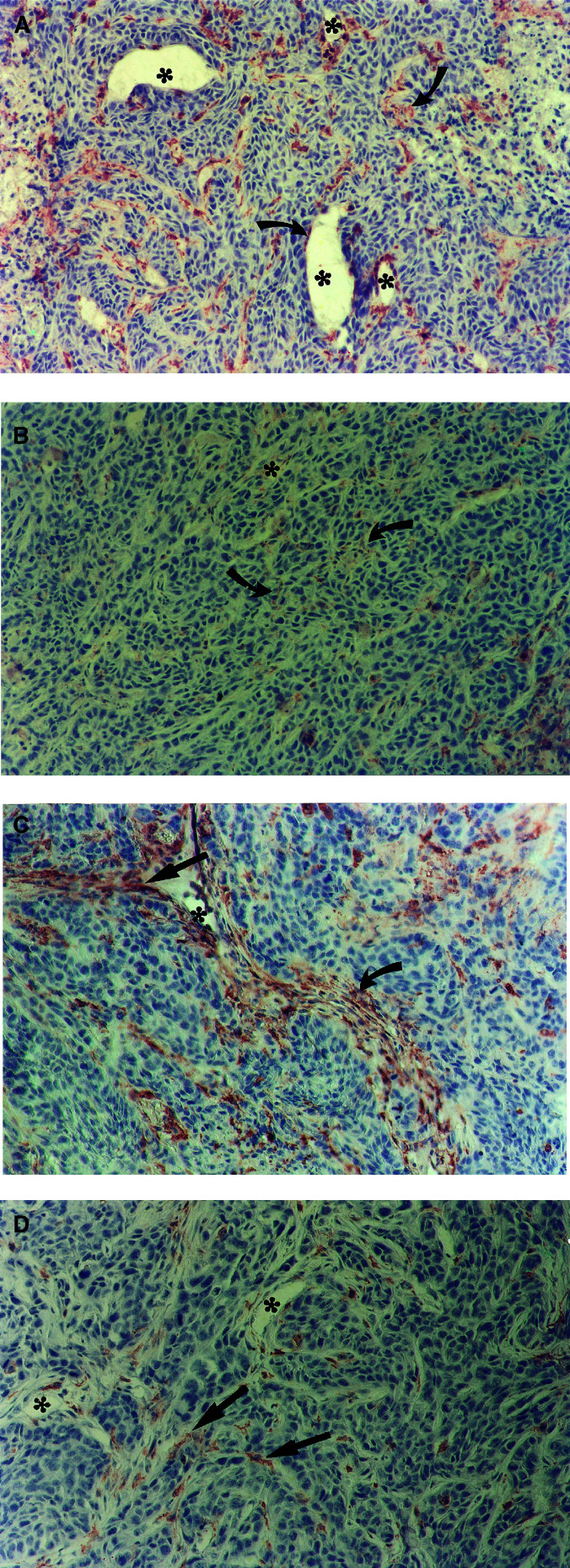
Effects of NaPaC on A431 tumour microvessel network. Endothelial cells were stained in early (**A**) and late (**C**) treatment controls, and in early (**B**) and late (**D**) NaPaC-treated tumours using GSL-1 lectin. Microvessel lumens in panels were indicated with asterisks. Magnification used was × 250. The representative AEC-stained endothelial cells (red) are indicated with arrows.

As we recently demonstrated that NaPaC inhibited *in vitro* the growth of human endothelial cells (HUV-EC) (Di Benedetto *et al*, 2002) and since we showed, in this paper above, that NaPaC competes with VEGF_165_ for the binding to endothelial cells, we evaluated the drug effects on microvessel development in A431 tumours (Figure 7

). We attempted to operate on vessel network in xenograft at two different stages of its formation by early (Figure 7B) and late (Figure 7D) administration of NaPaC.

**Figure 8:**
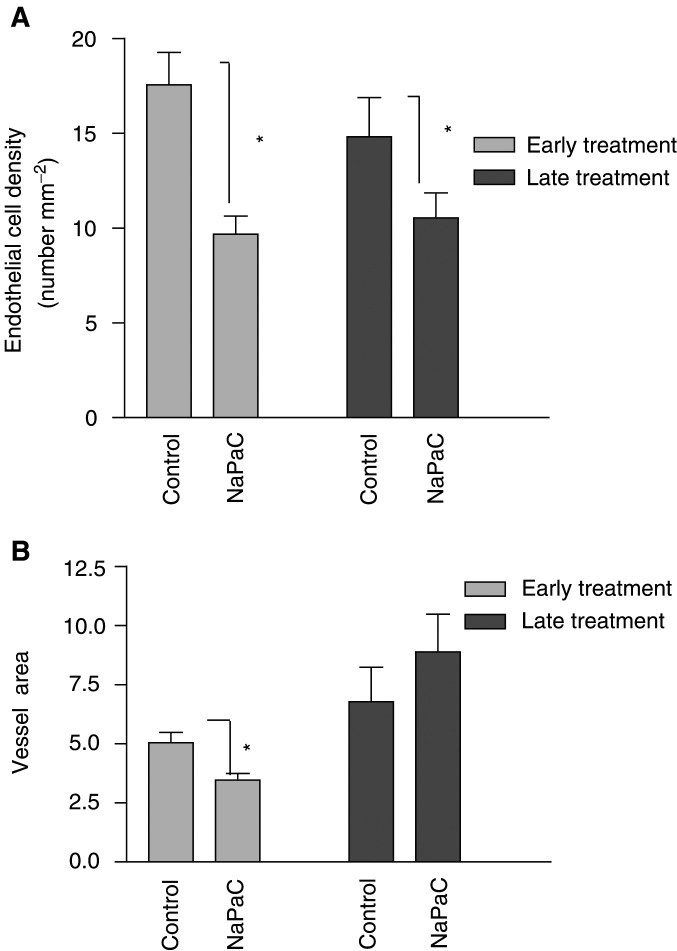
Quantification of endothelial cell density and vessel area in early and late NaPaC-treated tumours. (**A**) The GSL-1 lectin-stained endothelial cells per mm^2^ of tumour area (endothelial cell density) and (**B**) the fraction of the total tissue area occupied by the wall or/and lumen (vessel area) was determined as described in Materials and Methods. Each column represents the mean ± s.d. (*n*=10). ^*^P<0.05 *vs* control.

The number of endothelial cells per tumour tissue area (1 mm^2^) was decreased by 50% (*P*=0.006) after early NaPaC administration as compared to control (no treated) and 30% (*P*=0.045) after late treatment as compared to corresponding no treated control (Figure 8A

). When early treated tumours were compared to late treated ones this parameter was statistically similar. Concerning the fraction of the total tissue area occupied by the wall and/or lumen of vessel (vessel area), NaPaC was inefficient when used lately as compared to control (Figure 8B), whereas it has an inhibitory effect (35%, *P*=0.014) when injected early. Thus, NaPaC, administrated early, is able to affect the endothelial cell number and vessel area whereas NaPaC, injected late, alters only the first parameter.

## DISCUSSION

In this paper, we showed the antiproliferative, antiangiogenic and aponecrotic action of a new dextran derivative, NaPaC, on fast growing xenografts of A431 cells derived from an aggressive epidermoid carcinoma. A431 cells are known to secrete a large quantity of VEGF (Myoken *et al*, 1991), a potent angiogenic factor. We recently demonstrated that NaPaC interacted with VEGF_165_ by forming a complex and inhibited the proliferation of endothelial cells stimulated by VEGF_165_ (Di Benedetto *et al*, 2002). Here, we demonstrated, in addition, that NaPaC inhibited the binding of VEGF_165_ to its specific receptors on human endothelial cells. In the light of these NaPaC properties, we attempted to inactivate locally VEGF_165_ secreted by A431 cells at two different steps of xenograft development: by early administration of NaPaC, starting at tumour cell inoculation; and late treatment, beginning 1 week later when tumours were well established. Thus, we could operate on vessel network formation at two different stages. Since the tumour growth was largely demonstrated to be dependent on angiogenesis (Folkman, 1995; Carmeliet and Jain, 2000), we explored the impact of tumour vasculature evolution on the A431 xenograft growth.

In the case of both early and late treatments, NaPaC strongly inhibited the A431 tumour growth. It is well established now that tumour growth can be affected by tumour cell proliferation, tumour cell death and angiogenesis. Concerning cell proliferation, NaPaC was shown, here, to inhibit the *in vitro* A431 growth. This action could involve, at least in part, the decreasing VEGF_165_ binding to A431 cells as reported in this study. However, like Melnyk *et al* (1996), we were not able to evidence a VEGF dependence of A431 cell growth *in vitro* (data not shown) probably because of the high quantity of the secreted endogenous VEGF (Myoken *et al*, 1991). *In vivo*, we found that early NaPaC administration for 5 weeks was significantly more efficient than late one. Nevertheless, for both treatments, the A431 tumour uptake was observed at the same time after cell inoculation and the difference in growth rate of tumours only became significantly apparent after 4 weeks. In the light of these observations, the difference in effect of early and late NaPaC treatment cannot be explained considering only direct inhibitory effect of NaPaC on tumour cell proliferation.

In relation to tumour growth inhibition, we observed an increase in aponecrotic cell density in tumours. Indeed, the cell death was more important in early NaPaC-treated tumours than in late treated ones. Although, in our experimental conditions, we cannot distinguish the tumour and endothelial cells undergoing a death, it is clear that difference observed above is related to variations in the death of rather tumour cells than endothelial ones. The argument supporting this idea is that endothelial cell density was decreased in early and late treated tumours in the same manner. We recently reported that NaPaC induced *in vitro* the aponecrosis of breast cancer MCF-7ras cells (Di Benedetto *et al*, 2002) arguing for a possible direct aponecrotic effect of NaPaC on A431 cells. Nevertheless, *in vivo*, it is also likely that cell death was generated in tumour, at least in part, by oxygen deprivation of tissue owing to angiogenesis inhibition.

We showed in this report that both early and late treatments with NaPaC decreased, to the same extent, the endothelial cell density. In contrast, the vessel area, reflecting the overall number and/or size of vessels, was reduced in early treated tumours, whereas it was unchanged in late treated xenografts as compared to control. Thus, the vessel morphology in early and late treated tumours was different. These results showed that NaPaC, injected early, prevents the vessel enlargement and/or the increase in vessel number, these modifications being observed in late (1 week delayed) treated tumours as well as in control ones. Thus, a first week of A431 xenograft development, in the absence of NaPaC, is sufficient for morphological changes in intratumour vasculature. Interestingly, even 5 weeks NaPaC treatment was not able to affect these changes. The morphological transformations of intratumour vessels were recently described (Eberhard *et al*, 2001, Izumi *et al*, 2002, Leenders *et al*, 2002; Ryschich *et al*, 2002). In particular, it was observed that the early event of tumour angiogenesis consists in dilating the existing vessels prior to their sprouting (Eberhard *et al*, 2001; Leenders *et al*, 2002). This finding is in agreement with our observation that the vessel area was higher in late treated tumours, when NaPaC administration started 1 week after xenograft cell implantation, than in early treated ones, where NaPaC acted at the beginning of intratumour vasculature formation. As VEGF, produced in large amounts by A431 cells, has also vasodilating activity (Dvorak *et al*, 1999), it is possible that NaPaC administrated early was able to inactivate, at least in part, this growth factor and consequently to prevent vessel dilation. Since vessels are present even in the early treated tumours, it could be that A431 cells surround and co-opt, immediately after inoculation, the existing subcutaneous vessels as it was described in the case of non-small-cell-lung carcinoma (Pezzela *et al*, 1997) and melanoma (Leenders *et al*, 2002). Moreover, NaPaC seems to have no effect, administrated early or late, on this phenomenon. However, we cannot discard that in our experimental model the formation of neo-vessels occurs very early and that NaPaC is not able to inhibit it completely.

Altogether, our results showed that NaPaC inhibited the A431 tumour growth acting on both endothelial and tumour cells. The extent of this effect was dependent on the outset of NaPaC treatment. Since the period of NaPaC action on A431 cell proliferation was the same (5 weeks) and since the endothelial cell density was decreased in the same manner in both early and late treated tumours, the most probable is that the difference in tumour growth inhibition was because of changes in intratumour vascular network leading to the increase in tumour cell death observed above. Altogether, our data indicate that A431 xenograft model can be used to study the impact of vascular network in tumour growth and to screen potential antiangiogenic agents.

In conclusion, we demonstrated that NaPaC potently inhibits fast-growing epidermoid carcinoma by acting on tumour cells and intratumour endothelial cells whatever the state of xenograft development. Nontoxic at efficient doses, NaPaC provides interesting clues for therapies of solid tumours preventing the vascular network evolution in malignant lesions, thus inhibiting the rapid expansion from small tumours to late-stage tumours. Moreover, its direct inhibitory action on tumour cell proliferation argues for its usefulness in late-stage tumour treatment.
